# The Effects of Job Stress on Burnout and Turnover Intention: The Moderating Effects of Job Security and Financial Dependency

**DOI:** 10.3390/bs14040322

**Published:** 2024-04-12

**Authors:** Engin Üngüren, Neslihan Onur, Hüsne Demirel, Ömer Akgün Tekin

**Affiliations:** 1 Department of Business Management, Faculty of Economics, Administrative and Social Sciences, Alanya Alaaddin Keykubat University, Antalya 07450, Türkiye; engin.unguren@alanya.edu.tr; 2 Department of Gastronomy and Culinary Arts, Manavgat Faculty of Tourism, Akdeniz University, Antalya 07600, Türkiye; omeratekin@akdeniz.edu.tr; 3 Department of Social Work, Faculty of Health Sciences, Gazi University, Ankara 06490, Türkiye

**Keywords:** work stress, work-related burnout, turn-over intention, job security, financial dependence

## Abstract

(1) Background: The hospitality industry is known for exposing employees to work stress, which can lead to work-related burnout and high turnover rates. This study aims to examine the relationships between work stress, work-related burnout, and turnover intention. It also explores the mediating role of work-related burnout and the moderating role of job security and financial dependence. (2) Methods: A cross-sectional survey was conducted among 494 hotel employees working in five-star hotels in Belek and Manavgat, Türkiye, using a moderated mediation research model. The study found that work stress increases work-related burnout, which in turn increases turnover intention. Additionally, work-related burnout was found to mediate the relationship between work stress and turnover intention. Furthermore, it was found that perceived job security moderates the relationship between work stress levels and work-related burnout. Additionally, the variable of financial dependence was found to moderate the relationship between employees’ levels of work-related burnout and their turnover intentions. Similarly, the study found that the financial dependence variable moderates the effect of work-related burnout on employees’ turnover intention. Additionally, the study found that employees’ perception of job security moderates the impact of work stress on work-related burnout. In conclusion, the study suggests that positive perceptions of job security can mitigate the impact of work stress on work-related burnout. Similarly, the impact of work-related burnout on turnover intention diminishes as the degree of financial dependence rises.

## 1. Introduction

In the hospitality industry, employees are frequently exposed to work-related stress [1]. Work-related stress is a significant problem that incurs costs for both employees and businesses [2,3]. It is also known that hospitality industry employees face difficulties in coping with work-related stress [4]. Work stress is a significant challenge for the hospitality industry. Studies focusing on this problem have the potential to positively affect employees’ quality of life [5,6]. Therefore, work stress and related issues remain important research topics in the hospitality industry literature. Therefore, this study’s research model focuses on the problem of work stress and the variables it interacts with.

Work stress is a physiological, psychological, emotional, and behavioral reaction that occurs when the expectations in the work environment cannot be met with the needs, abilities, and resources of the employees [7,8]. This definition suggests that work stress is not a spontaneous phenomenon. Instead, various factors create favorable conditions for its emergence. Research has demonstrated that work stress can be caused by a variety of factors, including work overload, organizational, economic or technical change, bullying by co-workers [9], low organizational support, poor management practices [10], low pay policies, inadequate intra-organizational information sharing [11], leader guidance [12], home/work balance imbalance, workplace relationships, organizational climate, personal responsibility, and hassles at work [13]. Although work stress is a phenomenon that emerges under the influence of various factors, it also has negative consequences for employees and businesses [14,15]. Previous studies have shown that work stress levels can affect employees’ well-being [5], affective and normative commitment, job satisfaction [16], job performance [17], productivity [18], work alienation [19], depression, cardiovascular disease, cancer risks [20], turnover intentions [21], and burnout levels [22]. This study focuses on the work-related burnout problem, which is negatively affected by work stress, from among the aforementioned variables. This is because work-related burnout is a frequently observed issue among hospitality industry employees [23]. Jobs in the hospitality industry require constant contact with guests. Intensive interpersonal relationships throughout this process can lead to work stress and, consequently, work-related burnout [24]. Work stress negatively affects employees’ quality of life and health [25]. The World Health Organization defines quality of life as “individuals’ perception of their position in life in the context of the culture and value systems in which they live and in relation to their goals, expectations, standards and concerns” [26] (p. 1405). Furthermore, research has shown that work-related burnout, which is a significant consequence of work stress, has a negative impact on individuals’ quality of life [27,28,29].

The term ‘burnout’ was initially coined by American psychologist Herbert Freudenberger in the 1970s [30]. Maslach and Jackson [31], prominent researchers in the field of work-related burnout, have described it as a three-dimensional phenomenon consisting of emotional exhaustion, depersonalization, and reduced personal accomplishment. According to Maslach and Goldberg [32] “emotional exhaustion refers to feelings of being emotionally overextended and depleted of one’s emotional resources. Depersonalization refers to a negative, callous, or excessively detached response to other people, which often includes a loss of idealism. Reduced personal accomplishment refers to a decline in feelings of competence and productivity at work” (p. 64). Comprising a three-dimensional structure, work-related burnout and work stress are so closely linked that some researchers view work-related burnout as a unidimensional type of work stress [33]. Similarly, Maslach et al. [30] state that prolonged work stress is reported as one of the most significant causes of work-related burnout. Burnout is even defined as the end product of a chronic work stress process by Schaufeli and Enzmann [34]. Consistent with previous research, several studies [22,35,36,37,38,39] have found a positive correlation between work stress and work-related burnout. In line with these findings in the literature, the first hypothesis aims to determine the effect work stress has on work-related burnout. Therefore, the first hypothesis of this study is as follows:

**H_1_:**  Work stress positively affects work-related burnout.

Work-related burnout, considered a consequence of work stress in this study, negatively affects various work-related factors. Specifically, organizational commitment [40], job satisfaction [41], quality of service [42], and work performance [43] are negatively impacted by increasing levels of work-related burnout. In addition to these factors, work-related burnout levels can also negatively impact employees’ turnover intentions. In addition to these factors, work-related burnout levels can also negatively impact employees’ turnover intention. According to Maslach and Jackson [31] there is a negative correlation between work-related burnout and turnover intention. Turnover intention is a significant issue in the tourism sector, as well as in many other industries [44]. It can be referred to as “employees’ willingness or attempts to leave the current workplace voluntarily” [45] (p. 4). The labor-intensive nature of the hospitality industry limits the potential for automation [46], resulting in increased reliance on employees. Unpredictable employee turnover can cause significant damage to business operations. However, turnover can result in significant costs and losses for businesses, including loss of organizational knowledge, recruiting and training expenses for new employees [47], employee demoralization, loss of productive employees [48], and difficulties in finding replacements in the hospitality industry. Additionally, investments in departing employees such as training can be a burden for businesses. Therefore, finding ways to mitigate employees’ turnover intention is a crucial concern in hospitality industry research. Turnover intention is a significant predictor of actual turnover, and even the mere thought of leaving can have adverse effects on employees’ work attitudes and behaviors [49]. In this context, several studies in the literature [35,38,39,41,50,51,52] have evaluated work-related burnout as a factor that negatively affects employees’ turnover intention. Consistent with the literature, the second hypothesis of this study aims to investigate the impact of work-related burnout on turnover intention. The hypothesis is as follows:

**H_2_:**  Work-related burnout syndrome has a positive effect on turnover intention.

The presented hypotheses first focus on the impact of work stress on work-related burnout and then on the effect of work-related burnout on turnover intention. It is predicted that the effect of work stress on turnover intention is mediated through work-related burnout. As previously stated, work-related burnout is considered a result of chronic work stress experienced by employees [53]. Furthermore, work-related burnout, which is a consequence of work stress, is also recognized as a significant cause of turnover intention [54]. Several studies [55,56,57,58,59] have found that burnout mediates the relationship between work stress and turnover intention. Therefore, the third hypothesis of this study aims to determine the mediating role of work-related burnout in the effect of work stress on turnover intention. The hypothesis is presented as follows:

**H_3_:**  Work-related burnout mediates the relationship between work stress and turnover intention.

Another variable focused on in the study within the framework of the relationship between work stress and work-related burnout is job security. According to Joubert [60], job security is defined as “the perceived state in which an employee is secure within their job and does not fear to lose their job or any anticipated job features” (p. 26). Chronic problems in the hospitality industry, such as high-risk uncertainties, seasonal employment, and unpredictable changes in economic indicators, negatively affect employees’ perceived job security, which becomes an important work stress factor [61]. In addition, the hospitality industry was one of the sectors most affected by the COVID-19 pandemic in the recent past [62]. During this time, employees faced a major employment crisis, and their already problematic perceptions of job security were dramatically affected. In addition, the restructuring, institutionalization, and cost minimization efforts of hospitality businesses also cause employees to experience the problem of job security in a more challenging way [63]. For this reason, negative perceptions of job security, which is a very important factor in planning their future, can also negatively affect their attitudes and behaviors towards work [64]. In this context, from the opposite perspective, we anticipate that positive perceptions of job security may also buffer work stress levels. Previous studies [65,66,67,68] indicate that there is a negative relationship between employees’ perceived job security and work stress levels. This is because although working conditions in the hospitality industry have unique challenges, the fact that employees perceive the risk of losing their jobs as low can buffer their work stress levels [69]. When employees do not worry about losing their jobs, which are their sources of economic security, their work-related stress levels can be positively affected by this situation. However, it is possible to reach various findings demonstrating that there is a negative relationship between employees’ perceptions of job security and work-related burnout levels. In fact, with the opposite approach, job insecurity is seen as one of the most important factors causing employees to experience work-related burnout [70]. In this context, existing studies [70,71,72,73,74,75] have found that as perceived job insecurity increases, work-related burnout levels decrease. In this framework, although previous studies have revealed that work stress causes work-related burnout, we anticipate that this relationship may differ according to perceived job security. In other words, we think that the effect of work stress on work-related burnout can be buffered if employees’ perception of job security is positive. Based on these considerations, the fourth hypothesis of the study is presented:

**H_4_:**  Job security has a moderating role in the relationship between work stress and work-related burnout.

Regardless of the reason, work-related burnout, which is thought to occur as a result of various work stressors [10,13,24], is a factor that has significant effects on employees’ turnover intentions [39,52]. However, work-related burnout may not affect employees’ turnover intention to the same extent in all cases. At this point, employees’ level of financial dependency may play a moderating role in the relationship between work-related burnout and turnover intention. In other words, the effect of employees’ level of work-related burnout on their turnover intention may differ according to their level of financial dependency. It is possible to find different definitions of financial dependence in the literature [76,77]. However, in this study, the concept of financial dependence is accepted as the employee’s dependence on the economic returns of the job in exchange for their work in order to provide for themselves and their family. In other words, in this study, financial dependence includes employees who have no other source of income other than their job to sustain their economic security. In fact, financial dependence is an issue related to the inadequacy of income sources. Unemployment, unstable employment, or insufficient income to cover expenses negatively affect the financial dependence of workers [78]. However, the hospitality industry has conditions that negatively affect the financial dependence of workers in terms of the fact that such occupations generally do not require high qualifications [79]; these include seasonal working conditions [80], industry-specific risks that negatively affect employment [81], informal labor-intensive work, part-time employment, and lower wages [82]. Financially dependent employees experience the fear of being cut off from financial resources [83]. This fear due to financial dependency can lead to work stress [84]. For this reason, financial dependence may affect the decision-making process of employees in terms of quitting their jobs in a different way than many other issues. Therefore, the turnover intention of an employee with work-related burnout syndrome may differ according to the level of financial dependence. In other words, employees who do not have any financial resources other than their job to support themselves and their families may have a different attitude toward turnover behavior than employees who do have these resources. In this context, the fifth hypothesis of the study is presented as below:

**H_5_:**  Financial dependence plays a moderating role in the relationship between work-related burnout and turnover intention.

In the literature, it is possible to find many studies that examine the relationship between work stress, work-related burnout, and turnover intentions of employees in the hospitality industry. However, unlike previous studies, this study examines the relationships between job stress, work-related burnout, and turnover intentions in the context of the moderating roles of job security and financial dependency variables. In this regard, we believe that the results of this study will make specific contributions to the literature and practical applications. On the other hand, the variables in this study were analyzed based on the Conservation of Resources (COR) theory. COR theory is actually a work stress theory that reveals individuals’ assessment of the threat of losing their resources [15]. According to the COR theory, individuals try to develop and protect their resources because they need these resources in order to cope with work stress and to try to accumulate more resources [85]. Naturally, being able to survive economically thanks to the income they earn is one of the basic expectations of employees from an organization. In this context, job security and financial returns from work are important resources. Moreover, in the hospitality industry, seasonal work, sector-specific risks, informal labor-intensive work, part-time employment, and low wages [80,81,82] are factors that threaten resources and create work stress in the context of both job security and financial dependence. In this context, it is believed that the results of the research can be explained within the framework of COR theory. However, work stress [86], work-related burnout [87], turnover intention [88], job security [89], and financial conditions [90] are also factors that can affect employees’ quality of life. Therefore, the findings of this study are expected to contribute to the literature in the area of quality of life.

## 2. Materials and Methods

### 2.1. Sampling and Data Collection

This study was designed using quantitative methodology to evaluate the theoretical model and the hypotheses formulated based on this model. The cross-sectional design is the basis of this study, and the data were collected through a questionnaire survey. The study adopted a quantitative approach and targeted employees working in five-star hotels located in the tourism centers of Manavgat and Belek in Türkiye. Convenience sampling, one of the non-random sampling techniques, was used to reach the participants. Data collection was conducted between 9 January 2024 and 15 February 2024. During this period, employees of 12 different five-star hotels formed the sample of the study. During the selection of the participating hotels, managers, and general managers were contacted, informed about the objectives of the study and the data collection process, and their permission was obtained. The dates and methodology of data collection were agreed upon through interviews with these managers and their supervisors. The researchers collected the survey data by conducting one-on-one interviews with hotel employees between the agreed dates. During these interviews, the objectives of the study were explained in detail to the participants, and it was emphasized that participation was voluntary. Protecting the privacy of the participants was the primary goal; no identifying information was collected, and anonymity was ensured. This methodological approach aims to maximize the reliability of the research and the confidentiality of the participants. During the research process, 600 questionnaires were distributed. However, 106 questionnaires were deemed unsuitable for analysis due to their being incomplete or incorrectly filled out. After a thorough preliminary evaluation of the questionnaires, two main issues were identified. Firstly, a significant number of questionnaires were left blank. Secondly, more than one answer was given to some questions. Therefore, these questionnaires were excluded from the study to maintain the integrity of the analysis process and the quality of the data. As a result, only 494 fully and correctly completed questionnaires were included in the analysis phase. Regarding the gender distribution, 42.1% of the participants were women (*n* = 208) and 57.9% were men (*n* = 286). Overall, 39.1% of the participants were single (*n* = 193) and 60.9% were married (*n* = 301). The majority of participants, 65% (*n* = 319), were between 26 and 41 years old. In terms of educational level, high school graduates make up the largest proportion at 49.0% (*n* = 242), followed by primary education at 22.5% (*n* = 111), having an associate degree at 14.2% (*n* = 70), a bachelor’s degree at 11.7% (*n* = 58), and a master’s degree or higher at 2.6% (*n* = 13). When analyzing the distribution by area of work, food and beverage employees make up 32.2% (*n* = 159) of the total, while housekeeping employees make up the second largest group at 20.6% (*n* = 102). The proportion of employees working in the kitchen was 11.1% (*n* = 55), technical services were 9.9% (*n* = 49), reception was 10.3% (*n* = 51), accounting was 8.3% (*n* = 41), and other departments were 7.5% (*n* = 37). Regarding the length of time in the organization, 40.9% (*n* = 202) have been in the same job for 1–3 years, 27.3% (*n* = 135) for 3–5 years, 13.8% (*n* = 68) for 6–8 years, 12.1% (*n* = 60) for less than 1 year, and 5.9% (*n* = 29) for 9 years or more. This shows that the majority of participants have been in their jobs for short-to-medium-term periods.

### 2.2. Instrument

The questionnaire used in the study consists of six sections. The first part consists of the nine-item General Work Stress Scale developed by De Bruin [91], which examines the emotional, cognitive, motivational, and social consequences of the demands that individuals perceive at work and their interactions with these demands. All scale items were measured on a five-point Likert scale ranging from 1 = never; 2 = rarely; 3 = sometimes; 4 = often; to 5 = always. Higher scores indicate that participants experience high levels of job stress, while lower scores indicate lower levels of job stress. In the second part of the questionnaire, there is a six-item Job Security Perception Scale created by Gençdoğan Yılmaz and Aydın [92] to measure job security perceptions. This scale measures employees’ perceptions of job security in the organization, their concerns about arbitrary dismissal, their belief in job security, and their views on job security policies in the organization. Each item is rated on a five-point Likert scale ranging from 1 = strongly disagree to 5 = strongly agree. High scores on this scale indicate that employees have a high perception of job security, while low scores indicate that they perceive less job security. The third section is devoted to the work-related burnout scale developed by Kristensen et al. [93]. This scale measures the level of physical and psychological fatigue and exhaustion that individuals perceive in relation to their work. Scale items are measured on a five-point Likert scale ranging from 1 = never to 5 = always. Higher scores indicate that individuals experience high levels of work-related burnout, while lower scores indicate lower levels of burnout. The fourth section includes the three-item Financial Dependence Scale developed by Thompson et al. [94]. This scale assesses the role and importance of participants’ income from their jobs in providing for their families. Each item was rated on a five-point Likert scale ranging from 1 = strongly disagree to 5 = strongly agree. High scores indicate a high degree of financial dependence of the respondents on the income from their jobs. The fifth section includes a three-item scale adapted from Singh and Srivastava [95] to measure employee turnover intentions. Each item is rated on a five-point Likert scale ranging from 1 = strongly disagree to 5 = strongly agree. Higher scores on the scale indicate higher turnover intentions. The final section of the questionnaire was designed to collect demographic information about the participants. This section includes questions about basic demographic characteristics of the participants such as age, gender, education level, department they work in, and length of time they have been with the organization.

### 2.3. Data Analysis

**Figure 1:**
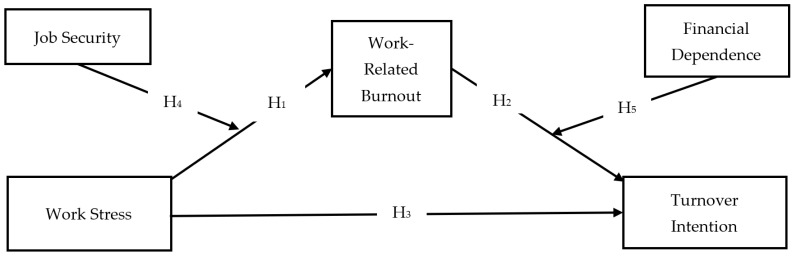
Conceptual model.

The moderated mediation research model examined in this study is designed based on the structure shown in Figure 1. This model shows how the mediation effect may differ in the presence of moderating variables, thus providing researchers with a more dynamic understanding of the relationships between variables. When preparing the data set for analysis, it is critical that it meets the requirements of multivariate statistical methods. In this regard, a detailed examination of missing values and potential outliers was conducted to assess whether the dataset met quality and reliability standards. Mahalanobis distance analysis was performed to identify outliers, and no outliers were found as a result of this analysis. Then, the normal distribution of the data was assessed by examining the skewness and kurtosis values. Skewness ranged from 1.47 to −0.25, while kurtosis spanned from 1.12 to −0.67. Based on Kline’s [96] criteria, these ranges indicate a normal distribution. This assessment supports the assumption of normality required for the statistical analyses used in this study. The analysis process of the model was carried out in two steps following the methodological framework proposed by Anderson and Gerbing [97]. The Anderson and Gerbing (1988) technique is widely accepted and used in various fields, such as psychology, management, marketing, and other social sciences, where SEM is commonly employed for theory testing and model evaluation [98,99,100,101]. This methodological approach distinguishes between the measurement model and the structural model. The measurement model assesses the reliability and validity of constructs, while the structural model tests hypothesized relationships between constructs. The measurement model was assessed in the first stage using confirmatory factor analysis (CFA). It determines the relationships between observed variables (indicators) and the corresponding latent variables (constructs). The purpose of Confirmatory Factor Analysis (CFA) is to evaluate the reliability and validity of the measurement model before proceeding to the structural model. In the second stage of the study, the process of testing the main hypotheses was carried out through the PROCESS macro model 1 and model 4 developed by Hayes [102]. Survey research can be affected by Common Method Bias (CMB) when respondents are asked to answer questions that involve both independent and dependent variables. To avoid this issue, the study utilized various procedural measures and statistical techniques [103]. As part of the procedural measures, participants received a comprehensive explanation of the study’s purpose; they were informed that participation was voluntary, and that they could withdraw at any time. Additionally, they were assured that their responses would remain confidential [104]. This approach aims to reduce common method bias by minimizing the likelihood of respondents altering their answers due to social desirability or perceived expectations from others. Statistical analysis was conducted to assess the risk of CMB using Harman’s single-factor approach [104]. Exploratory factor analysis (EFA) was applied to the propositions in the research model without using any rotation techniques, with the criterion of an eigenvalue greater than 1. The exploratory factor analysis (EFA) resulted in five factors that accounted for 72.42% of the variance. The first factor explained 25.45% of the variance, which is below the threshold value of 50%. Therefore, it can be concluded that CMB is not a significant issue.

## 3. Results

### 3.1. Assessment of the Measurement Model

The results of the confirmatory factor analysis (CFA) of the measurement model developed based on the theoretical framework underlying the research are presented in Table 1. This model includes five separate theoretical constructs and a total of 28 items expressing these constructs. According to the CFA results, the standardized factor loadings for each of the scale items are above 0.50 and have statistically significant t-values. The goodness of fit of the model (χ^2^ = 633,287; DF = 335. χ^2^/df = 1.89; RMSEA = 0.042; SRMR = 0.026; NFI = 0.961; RFI = 0.956; IFI = 0.981; CFI = 0.981) indicates that the measurement model has an adequate structure [105]. Cronbach’s alpha reliability coefficients ranging from 0.93 to 0.97 indicate that the theoretical constructs discussed in the study have a high degree of internal consistency [106].

### 3.2. Convergent Validity and Discriminant Validity

The results of convergent validity, discriminant validity, and construct reliability of the research model are presented in Table 2. In the convergent validity evaluation, the CR and AVE values were examined. CR expresses the consistency among scale items of a conceptual construct, and values above 0.70 are considered sufficient [107]. In the table, the CR values for each construct ranged from 0.93 to 0.97, indicating that the scales were highly reliable. The AVE indicates the average amount of variance explained by the scale items of a construct, and values of 0.50 or greater indicate adequate convergent validity [107]. The AVE values in Table 2 range from 0.71 to 0.87, indicating that each construct has convergent validity. The fact that the CR values of all constructs are greater than 0.70 and the AVE values are greater than 0.50, and that the CR values are also greater than the AVE values, supports the convergent validity of the constructs. Discriminant validity, on the other hand, indicates how well a measure can distinguish a particular concept from other concepts. According to the criteria proposed by Fornell and Larcker [108], the square root of the AVE value of a construct should be greater than its correlations with other concepts. In Table 2, it is observed that the square root of the AVE of each construct is greater than the correlations of the construct with other concepts. In addition, the MSV is smaller than the AVE, and the MaxR(H) values are greater than 0.85, indicating that the measured constructs are sufficiently discriminated from each other. In this study, the MSV values for all constructs are lower than the corresponding AVE values, indicating that discriminant validity is strongly established. As a result, the results of the analysis in Table 2 show that the measurement model under study has achieved convergent validity and discriminant validity.

Table 3 presents a comparison of different measurement models for the main conceptual constructs of the research model. This comparison allows the evaluation of five different models (five-, four-, three-, two-, and one-factor models) based on statistical fit indicators such as chi-square (χ^2^), degrees of freedom (df), χ^2^/df, CFI, SRMR, and RMSEA. The hypothesized five-factor model was found to have the highest fit indicators. The analysis of the alternative models shows that the fit indicators decrease significantly in these models. Specifically, χ^2^ values increase, CFI values decrease, and RMSEA values increase as model complexity decreases. This suggests that the hypothesized five-factor model fits the data set better than the other models. This comparative analysis indicates that considering the conceptual structures of the research model separately is most appropriate for the data. The comparison with alternative models supports that the structure of the model requires that the conceptual constructs be treated independently of each other, and that this independence is statistically significant. Therefore, it is concluded that the five-factor model accurately represents the conceptual constructs addressed in the research and effectively reflects the relationships among these constructs.

### 3.3. Hypothesis Test

Table 4 presents the results of the mediation analysis, which shows the mediating role of burnout (BRNT) in the relationship between job stress (WSTRS) and turnover intention (TRNINT). As a result of this analysis, the effect of job stress on burnout, the effect of burnout on turnover intention, and both direct and indirect effects of job stress on turnover intention through burnout were evaluated. According to the results in Table 4, job stress has a positive effect on burnout (β = 0.40, t(492) = 14.98, %95 CI [0.34; 0.45], *p* < 0.001). This suggests that as job stress increases, so does burnout. The direct effect of job stress on turnover intention (β = 0.32, t(491) = 7.56, %95 CI [0.23; 0.40], *p* < 0.001) and the effect of burnout on turnover intention (β = 0.40, t(491) = 6.78, %95 CI [0.29; 0.52], *p* < 0.001) were also significant and positive. The indirect effect of job stress on turnover intentions through burnout (β = 0.16, %95 BCA CI [0.11; 0.21]) was statistically significant. These results suggest that an increase in job stress leads to a direct increase in turnover intention and, at the same time, indirectly increases turnover intention by increasing burnout levels. This suggests that the enhancing effect of job stress on turnover intention is realized both directly and through burnout. Therefore, burnout plays a partial mediating role in the relationship between job stress and turnover intention, which partially explains the relationship between these two variables. According to these results, hypotheses H_1_, H_2_, and H_3_ are supported.

**Figure 2:**
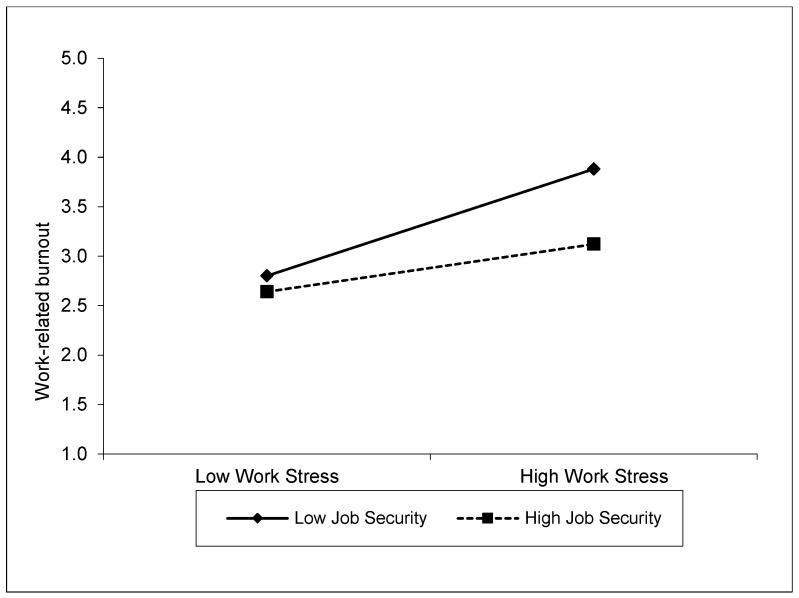
Moderating effect of job security on the relationship between work stress and work-related burnout.

Table 5 presents the results of the moderation analysis, which tests the moderating effect of job security on the relationship between job stress and burnout. This analysis shows how the effect of job stress on burnout varies with the level of job security. According to the results of the analysis, the effect of job stress on burnout is positive (β = 0.39, SE = 0.02, t = 17.76, %95 CI [0.34, 0.43], *p* < 0.001). Job security also has a negative effect on burnout (β = −0.23, SE = 0.02, t = −12.46, %95 CI [−0.27, −0.20], *p* < 0.001). This suggests that employees with high job security experience lower levels of burnout. At the same time, the interaction of job stress and job security (WSTRS × JOBSEC) shows a significant moderating effect on burnout (β = −0.15, SE = 0.02, t = −8.86, %95 CI [−0.18, −0.11], *p* < 0.001). This finding suggests that the effect of job stress on burnout decreases when job security is high. When the conditional effects of job security were analyzed, the effect of job stress on burnout was stronger for individuals with low job security (β = 0.57, SE = 0.03, t = 18.05, %95 CI [0.50, 0.63], *p* < 0.001). This effect was weaker for individuals with high job security (β = 0.18, SE = 0.03, t = 5.81, %95 CI [0.12, 0.26], *p* < 0.001). These results suggest that job security plays an important moderating role in the relationship between job stress and burnout. Situations where job security is high mitigate the negative effects of job stress and reduce individuals’ burnout levels. These results are visualized in Figure 2. According to this result, hypothesis H_4_ is supported.

**Figure 3:**
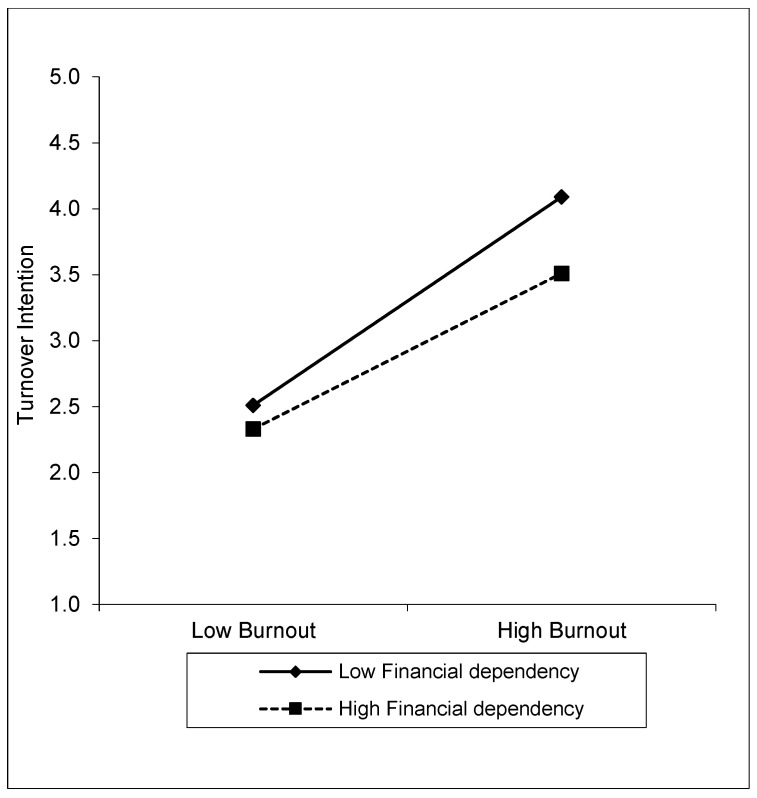
Moderating effect of financial dependence on the relationship burnout and turnover intention.

The results in Table 6 show the moderating effect of financial dependency on the relationship between burnout and turnover intention. According to the analysis results, burnout has a positive effect on turnover intention (β = 0.69, SE = 0.05, t = 13.74, %95 CI [0.59, 0.79], *p* < 0.001). Financial dependence has a negative effect on turnover intention (β = −0.19, SE = 0.03, t = −6.07, %95 CI [−0.25, −0.13], *p* < 0.001). This result indicates that employees with higher levels of financial dependency have lower turnover intentions. However, the interaction of burnout and financial dependence (BRNT × FINDEP) shows a significant moderating effect on turnover intentions (β = −0.10, SE = 0.04, t = −2.53, %95 CI [−0.18, −0.02], *p* < 0.001). This finding suggests that the effect of burnout on turnover intention is reduced when financial dependence is high. The effects of financial dependence as a moderator variable are shown in Figure 3. Considering the conditional effects of financial dependence, the effect of burnout on turnover intention is stronger for employees with low financial dependence (β = 0.85, SE = 0.08, t = 10.39, %95 CI [0.69, 1.01], *p* < 0.001). This effect was weaker for employees with high financial dependence (β = 0.54, SE = 0.07, t = 7.22, %95 CI [0.40, 0.69], *p* < 0.001). These results suggest that financial dependence plays an important moderating role in the relationship between burnout and turnover intention. High financial dependence acts as a buffer in reducing individuals’ turnover intentions and mitigates the impact of burnout in this process. These findings suggest that hypothesis H_5_ is supported.

## 4. Discussion

Work stress and the resulting work-related burnout can have devastating consequences and high costs for organizations and employees in the hospitality industry [109]. In this context, the study first aimed to examine the relationships between job stress and work-related burnout in accordance with hypothesis H_1_. As a result of the analysis, it was found that job stress has a negative effect on the level of work-related burnout among employees. This finding is consistent with the results of previous studies. In fact, many studies present work-related burnout as a direct result of work stress [110]. Additionally, Chiang and Liu [35], focusing on housekeeping department employees in Taiwan, found that work stress increased the level of work-related burnout among employees. They also reported that repetitive job content and intense workload also cause employees to experience work stress, which affects work-related burnout. Similarly, Jung and Yoon [36], in their study on employees in the hospitality industry in Korea, found that there is a positive relationship between work stress and employees’ work-related burnout levels. In their study, they also found that work stress caused by factors such as irregular working hours, long hours and overwork, low wages, and direct contact with customers in the hospitality industry increases burnout. Koc and Bozkurt [22], in their research on hotel employees in Türkiye, addressed the issue with a different approach. In this study, they found that employees who expect the current level of job stress to increase in the future have higher burnout symptoms. They also found that the repetition of the failures experienced by the employees or the increase in their perception of the threat to their jobs causes work stress and, consequently, burnout. As a result of the study conducted by Salama et al. [39] with hotel employees in Egypt, it was found that there is a positive relationship between work stress and work-related burnout. In this study, it was found that job stress is the main reason why employees experience work-related burnout.

According to hypothesis H_2_, the relationship between work-related burnout and turnover intention was analyzed. As a result of the analysis, it was found that as the level of work-related burnout of employees increases, their turnover intention also increases. This finding is consistent with the results of previous studies, namely that turnover is a global problem in the hospitality industry. In fact, the hospitality industry is in a more unfavorable situation than many other sectors in terms of the turnover problem [111]. It is estimated that employee turnover can cost about 1.5 times more than their annual salary. In other words, turnover is actually a serious problem for companies economically [48]. Chiang and Liu [35] stated that work-related burnout is a factor that triggers turnover, and turnover is known to be an important issue in the hospitality industry. According to Salama et al. [39], the challenges arising from the working conditions in the hospitality industry increase the level of work stress and work-related burnout among employees. As a result, over time, there is a noticeable decline in performance and, in the final stage, it is understood that employees decide to quit their jobs. In a study conducted by Pu et al. [41] with employees in the hospitality industry in China, it was found that employees in high work intensity and low wage conditions experience emotional exhaustion, which increases turnover intentions. Similarly, a study conducted by Park and Kim [112] on employees at five-star hotels in Seoul found that work-related burnout increased turnover intentions. In the related study, it was found that the emotional labor performance of hotel employees causes work stress and burnout, and the increasing level of work-related burnout leads employees to quit their jobs. Xing et al. [52], in their study on students undergoing internships in hotels in China, stated that the intense work stress and work-related burnout experienced by the employees, especially in frontline departments, caused burnout and as a result, the employees preferred to leave the hospitality industry. Demirdağ et al. [50], in their study conducted with hotel industry employees in Türkiye, concluded that work-related burnout levels of employees increase their turnover intentions.

In line with hypothesis H_3_, the mediating role of work-related burnout in the relationship between work stress and turnover intention was examined. As a result of the analysis, it was found that the effect of work stress on turnover intention is mediated by work-related burnout. This finding is consistent with the findings of previous studies. Ahmad and Afgan [55] conducted a study on bank employees in Pakistan and found that there was a positive relationship between work stress and turnover intention. In addition, work-related burnout was found to play a partially mediating role in the relationship between work stress and turnover intention. In a study conducted by Kim and Stoner [57] on social workers in the USA, it was found that high levels of job stress increase burnout and, as a result, increase the likelihood of social workers leaving their jobs In this study, burnout was found to play a mediating role in the relationship between job stress and turnover intentions. In a study conducted by Wen et al. [59] on frontline hotel employees in China, it was found that employees who experienced work stress did not quit their jobs immediately unless they experienced high levels of work-related burnout. In this regard, work-related burnout was found to fully mediate the relationship between job stress and turnover intentions.

In the context of hypothesis H_4_, the study examined whether job security plays a moderating role in the relationship between job stress and work-related burnout. The acquired results indicated that the effect of job stress on work-related burnout differed according to participants’ perceptions of job security. In other words, it was found that the effect of job stress on work-related burnout was buffered by job security. Although employees are exposed to high levels of job stress, the fact that they perceive their job security to be high in terms of maintaining their economic security buffers the effect of job stress levels on work-related burnout. From the opposite point of view, the fact that employees who are already exposed to high levels of job stress also perceive the risk of losing their job as high may cause them to experience more burnout. On the other hand, several studies in the literature have found that there is a negative relationship between job stress and job security, i.e., as job security increases, the level of job stress decreases. In a study conducted by Soelton et al. [66] in a technology company in Indonesia, it was found that the level of job stress increased as employees’ perceived job security decreased. Similarly, in a study conducted by Suhaimi [67] on teachers in Malaysia, a negative relationship was found between work stress and job security. Accordingly, a decrease in perceived job security increases the existing work stress, while an improvement in perceived job security buffers the existing work stress. There are also negative relationships between job security and work-related burnout in the literature. Aybas and Dündar [71] conducted a study on white-collar employees in Türkiye and found that there is a positive relationship between job insecurity and burnout. Çetin and Çolak [73] conducted a study on teachers in Türkiye and found that as teachers’ job insecurity increased, their work-related burnout decreased. Blom et al. [72] study on employees in Sweden also found that as employees’ job insecurity increased, their burnout also increased. The fact that there is a negative relationship between the job security variable and work stress and work-related burnout helps to better understand that the effect of work stress on burnout is buffered by job security.

In the last hypothesis of the study, the moderating role of financial dependence in the relationship between work-related burnout and turnover intention was examined. As a result of the analysis, the effect of work-related burnout on turnover intention decreases as the level of financial dependence of employees increases, and it is determined that financial dependence plays a moderating role in this relationship. This finding actually reveals a dramatic result: even if employees who have no financial resources other than the income from their job experience high levels of work-related burnout, their turnover intentions are affected at a lower level. On the other hand, the effect of work-related burnout levels on turnover intentions is stronger for those who feel less financially dependent. In this context, it is understood that employees who have no economic income other than their job and who are in economically difficult conditions are more patient in their turnover intentions, even if they experience high levels of work-related burnout. This suggests that as employees’ economic dependence decreases, their turnover intentions increase, i.e., an improvement in employees’ economic conditions may trigger them to leave their jobs. Similarly, in a study conducted by Thompson et al. [94] on working mothers, it was found that there was a negative relationship between financial dependency and turnover. In other words, it was found that as participants’ financial dependency increased, their turnover intentions decreased. In this context, employees who have to say “I need this job” due to the economic difficulties they are experiencing resist all difficulties in order not to leave the job they have. This finding is believed to reflect the reality of employees working under difficult conditions.

We believe that the results of the research can be explained by the COR theory. COR theory is a work stress theory based on the premise that employees try to protect, renew, and develop their resources and that they experience work stress when they fail to do so [15]. According to this theory, employees’ feelings of lack of job security and high financial dependency can lead to an increase in work stress. We believe that the results obtained in this framework also contribute to the field of COR theory; the results revealed that there are negative relationships between positive perceptions of an important resource such as job security and work stress and work-related burnout. In other words, when employees have positive thoughts about job continuity, which is an important resource for economic security, their levels of work stress and work-related burnout decrease. On the contrary, increasing the risk of losing the source of job security negatively affects work stress and work-related burnout.

Work stress and work-related burnout are also factors that can negatively affect the quality of life of employees in psychological terms [113,114]. The concepts of job security and financial dependence affect the quality of life of employees more in the context of economic living conditions [115]. However, it is inevitable that these factors affecting economic security also have psychological consequences. This is because limited economic opportunities and the resulting disadvantages are known to cause stress and negative emotional states [116]. Work stress and work-related burnout are effective in reducing the quality of life of individuals in terms of worrying problems such as anxiety, depression, irritability, fatigue, withdrawal, aggression, sadness, low motivation, palpitations, nausea, headaches, and cardiovascular diseases [117,118]. In addition, the fact that employees have no job security and are financially dependent only on the resources they receive from their own work causes them to feel anxious, depressed, suffer from insomnia, have health problems due to the decrease in their psychological well-being, and their quality of life is negatively affected [89,119,120]. It should not be overlooked that it is extremely difficult for an employee living under these conditions to perform at a sufficiently high level, to be loyal to the organization, and to be a productive employee. Therefore, helping hospitality workers cope with job stress, improving their perceptions of job security, and taking steps to improve their financial conditions will reduce the impact of these problems on their quality of life.

## 5. Conclusions

As a result of the study, consistent with the findings of previous studies, it was found that job stress has a negative effect on work-related burnout and that work-related burnout increases turnover intentions of employees. However, unlike previous studies, this study also examined the moderating role of job security and financial dependency in this relationship. As a result of the analyses, it was determined that the effect of job stress levels on work-related burnout can be buffered if job security perceptions are positive. In other words, it was found that the effect of job stress on work-related burnout decreases as employees’ concerns about losing their jobs decrease. On the other hand, it was also found that the effect of work-related burnout on turnover intentions decreased as employees’ financial dependency increased. In other words, even if the level of work-related burnout is high among employees who have no income other than their job to sustain their economic security, this situation does not effectively cause turnover intention. It is believed that these findings obtained from the research can contribute to the gap in the literature. On the other hand, it is anticipated that the results can provide a different perspective for practical applications. It is predicted that combating work stress, which is a dependent variable in this research model, will reduce both work-related burnout levels and turnover intentions of employees. In addition, it is known that actively combating work stress is a step that reduces the costs of organizations [5]. The fact that employees are not left alone in the struggle against work stress and the development of organizational struggle systems at this point can significantly affect success [121]. In particular, measures to remedy this issue include working on practices that increase the organizational commitment of employees [35], improving the conditions that can cause stress in the work environment, developing training programs, organizing cultural and recreational activities [36], active support from the managers in the process [39,50], and following an empowerment policy to support employees in specific areas [1,122]. Despite all these measures, work stress is a phenomenon that cannot be completely eliminated. Therefore, reducing the problem of work stress to a level that can be managed as much as possible can improve the quality-of-life conditions of employees in general and their work life in particular. The results of the study show that employees in economically challenging conditions are more resilient in terms of their tendency to quit their jobs, even though they experience high levels of work-related burnout. However, it is questionable how beneficial it is for the other members of the organization and the company that employees in these conditions need to continue in their current jobs. It should not be overlooked that employees who are constantly experiencing work stress and intend to quit their jobs may intentionally or unintentionally harm the organization and their colleagues.

## 6. Theoretical and Practical Implications

This study offers both theoretical and practical implications for researchers and industry professionals working in the field of hospitality industry. The findings obtained in the research revealed that, theoretically, work stress increases employees’ turnover intention through work-related burnout. In other words, work-related burnout was determined to be a remarkable mediating variable in the relationship between work stress and turnover intention. This finding theoretically reveals that work-related burn-out is a critical variable in the relationship between work stress and turnover intention. However, when the relationship between work stress, work-related burnout and turnover intention was considered in the context of employees’ job security perceptions and financial dependence levels, it was determined that the interaction between the variables differed. More precisely, revealing that improving employees’ perception of job security can be a strategic factor in buffering the effect of work stress on turnover intention is seen as a remarkable contribution to the theoretical field. In addition, another striking finding that we think may contribute to the theoretical field is the role of financial dependence in this relationship. Our results showed that the decrease in employees’ financial dependence levels also increases the effect of work stress on turnover intention.

From a practical perspective, this study offers meaningful implications, especially for hospitality industry practitioners. According to the findings, improving employees’ perception of job security reduces the negative effect of work stress on work-related burnout. Due to the working conditions in the hospitality industry, it may not be possible to completely eliminate work stress in practice. However, in order to reduce the impact of work stress on work-related burnout, employment policies can be implemented to improve employees’ perception of job security. In addition, providing awareness-raising training and implementing social activity programs can be turned into a human resources policy in order to reduce employees’ work stress levels and cope with work-related burnout.

## 7. Recommendations for Future Researchers

In this study, the mediating role of work-related burnout in the relationship between work stress and turnover intention was examined. This is because work-related burnout is a common syndrome among employees in the hospitality industry. However, in future studies, the roles of different mediator variables in the relationship between these two variables can be examined. On the other hand, in this research, the moderating role of the job security variable, which is a chronic problem in the hospitality industry, in the relationship between work stress and work-related burnout was examined. However, future researchers may particularly focus on the role of chronic problems such as seasonality effect and part-time employment. Additionally, examining the financial well-being variable in the relationship between work-related burnout and turnover intention in future studies may offer a different perspective to the literature. Finally, this research was carried out in hotels serving the sea–sand–sun tourism concept. However, future research can take a different approach by focusing on the employees of city hotels.

## 8. Limitations

The results of the research should be evaluated within the limitations of the research. The cross-sectional method was used in the study due to the difficulties of data collection in the field. However, it may be useful to prefer longitudinal methods in future studies to obtain more comprehensive and generalizable results. Also, convenience sampling was preferred among the sampling methods within the research capabilities. While this method provides a quick and economical way to collect data, it also requires caution in generalizing the results. For this reason, it is believed that it may be more beneficial for future researchers to prioritize probability sampling methods. Finally, only quantitative methods were used in this study. However, the preference of future researchers for mixed methods may contribute to the emergence of more in-depth findings.

## Figures and Tables

**Table 1:** Results of the measurement model.

Construct	Items	Factor Loadings	S.E.	t Values	*p*	Cronbach Alpha
Work Stress	WSTRS 1	0.916		Fixed		0.97
	WSTRS 2	0.796	0.037	22.039	<0.001	
	WSTRS 3	0.821	0.035	26.636	<0.001	
	WSTRS 4	0.818	0.032	26.342	<0.001	
	WSTRS 5	0.878	0.029	31.292	<0.001	
	WSTRS 6	0.884	0.028	31.859	<0.001	
	WSTRS 7	0.899	0.028	33.361	<0.001	
	WSTRS 8	0.932	0.024	37.065	<0.001	
	WSTRS 9	0.953	0.023	39.993	<0.001	
Job Security	JOBSEC 1	0.947		Fixed		0.97
	JOBSEC 2	0.863	0.030	31.780	<0.001	
	JOBSEC 3	0.905	0.026	37.596	<0.001	
	JOBSEC 4	0.883	0.028	34.307	<0.001	
	JOBSEC 5	0.920	0.023	39.655	<0.001	
	JOBSEC 6	0.951	0.020	45.440	<0.001	
Work-Related Burnout	BRNT 1	0.857		Fixed		0.94
	BRNT 2	0.763	0.056	17.863	<0.001	
	BRNT 3	0.807	0.043	22.610	<0.001	
	BRNT 4	0.831	0.045	23.964	<0.001	
	BRNT 5	0.867	0.039	25.834	<0.001	
	BRNT 6	0.878	0.039	26.266	<0.001	
	BRNT 7	0.891	0.038	27.086	<0.001	
Financial Dependence	FINDEP 1	0.940		Fixed		0.95
	FINDEP 2	0.874	0.030	32.661	<0.001	
	FINDEP 3	0.977	0.022	46.135	<0.001	
Turnover Intention	TRNINT 1	0.931		Fixed		0.93
	TRNINT 2	0.842	0.031	28.012	<0.001	
	TRNINT 3	0.945	0.026	37.462	<0.001	

**Table 2:** Convergent validity and discriminant validity.

	CR	AVE	MSV	MaxR(H)	1	2	3	4	5
1. WSTRS	0.97	0.77	0.34	0.98	[0.88]				
2. JOBSEC	0.97	0.83	0.17	0.97	−0.11 *	[0.91]			
3. BRNT	0.95	0.71	0.34	0.95	0.56 **	−0.47 **	[0.84]		
4. FINDEP	0.95	0.87	0.03	0.97	0.03	0.04	0.12 **	[0.93]	
5. TRNINT	0.93	0.82	0.27	0.95	0.51 **	−0.30 **	0.49 **	−0.17 **	[0.91]

AVE: Average variance extracted; CR: construct reliability; BRNT: work-related burnout; FINDEP: financial independence; JOBSEC: job security; MSV: maximum shared variance; TRNINT: turnover intention; WSTRS: work stress; values in square brackets [ ] are the square root values of AVE, * *p* < 0.05, ** *p* < 0.01.

**Table 3:** Comparison of alternative measurement models for the main constructs.

Modes	χ^2^	df	χ^2^/df	CFI	SRMR	RMSEA		Model Comparison		
								∆χ^2^	∆df	*p* (∆χ^2^)
1. Hypothesized five-factor model ^a^	633.287	335	1.89	0.98	0.026	0.042		-	-	
2. Two-factor model ^b^	3149.18	334	9.155	0.82	0.148	0.129	2 vs. 1	2515.89	1	*p* < 0.001
3. Three-factor model ^c^	6924.02	347	19.954	0.58	0.221	0.196	3 vs. 1	6290.731	12	*p* < 0.001
4. Two-factor model ^d^	7937.6	349	22.744	0.52	0.225	0.210	4 vs. 1	7304.312	14	*p* < 0.001
5. One-factor model ^e^	9515.5	350	27.187	0.42	0.237	0.230	5 vs. 1	8882.215	15	*p* < 0.001

^a^ = Work stress; job security; work-related burnout; financial dependence; turnover Intention. ^b^ = Work stress + work-related burnout; job security; financial dependence; turnover intention. ^c^ = Work stress + work-related burnout + job security; financial dependence; turnover intention. ^d^ = Work stress + work-related burnout + job security + turnover intention; financial dependence. ^e^ = work stress + work-related burnout + job security + turnover intention and financial dependence.

**Table 4:** Results of mediation analysis.

	Mediator (BRNT)					Dependent (TRNINT)				
Antecedent	β	SE	t Statistic	LLCI	ULCI	β	SE	t Statistic	LLCI	ULCI
WSTRS	0.40	0.03	14.98	0.34	0.45	0.32	0.04	7.56 ***	0.23	0.40
BRNT	-	-	-	-	-	0.40	0.06	6.78 ***	0.29	0.52
	R^2^ = 0.31 F_(1,492)_ = 224.51, *p* < 0.001					R^2^ = 0.32 F_(2,491)_ = 116.81 *p* < 0.001				
Total effect of WSTRS → TRNINT						0.48	0.04	13.11 ***	0.40	0.55
Direct effect WSTRS → TRNINT						0.32	0.04	7.56 ***	0.23	0.40
Bootstrap Indirect Effects WSTRS → BRNT → TRNINT						0.16	0.03	-	0.11	0.21

WSTRS: Work stress, BRNT: work-related burnout, TRNINT: turnover intention; LLCI: lower limit confidence interval; ULCI: upper limit confidence interval; Bootstrap sample size = 50,000; *** *p* < 0.001.

**Table 5:** Result of moderating effect of job security.

	Dependent (BRNT)				
Antecedent	β	SE	t Statistic	LLCI	ULCI
WSTRS	0.39	0.02	17.76 ***	0.34	0.43
JOBSEC	−0.23	0.02	−12.46 ***	−0.27	−0.20
WSTRS × JOBSEC	−0.15	0.02	−8.86 ***	−0.18	−0.11
	R^2^ = 0.55 F_(3,490)_ = 199.72, *p* < 0.001				
Conditional effects of JOBSEC	β	SE	t statistic	LLCI	ULCI
Low JOBSEC: WSTRS → BRNT	0.57	0.03	18.05 ***	0.50	0.63
High JOBSEC: WSTRS → BRNT	0.18	0.03	5.81 ***	0.12	0.26

WSTRS: work stress, JOBSEC: job security, BRNT: work-related burnout, LLCI: lower limit confidence interval; ULCI: upper limit confidence interval; Bootstrap sample size = 50,000; *** *p* < 0.001.

**Table 6:** Result of moderating effect of financial dependence.

	Dependent (TRNINT)				
Antecedent	β	SE	t Statistic	LLCI	ULCI
BRNT	0.69	0.05	13.74 ***	0.59	0.79
FINDEP	−0.19	0.03	−6.07 ***	−0.25	−0.13
BRNT × FINDEP	−0.10	0.04	−2.53 ***	−0.18	−0.02
	R^2^ = 0.30 F_(3,490)_ = 71.17, *p* < 0.001				
Conditional effects of FINDEP	β	SE	t statistic	LLCI	ULCI
Low FINDEP: BRNT → TRNINT	0.85	0.08	10.39 ***	0.69	1.01
High FINDEP: BRNT → TRNINT	0.54	0.07	7.22 ***	0.40	0.69

BRNT: burnout, FINDEP: financial dependence, TRNINT: turnover intention, LLCI: lower limit confidence interval; ULCI: upper limit confidence interval; Bootstrap sample size = 50,000; *** *p* < 0.001.

## Data Availability

The data that support the findings of this study are available from the corresponding author upon reasonable request.
